# Remdesivir and GS-441524 Retain Antiviral Activity against Delta, Omicron, and Other Emergent SARS-CoV-2 Variants

**DOI:** 10.1128/aac.00222-22

**Published:** 2022-05-09

**Authors:** Jared Pitts, Jiani Li, Jason K. Perry, Venice Du Pont, Nicholas Riola, Lauren Rodriguez, Xianghan Lu, Chaitanya Kurhade, Xuping Xie, Gregory Camus, Savrina Manhas, Ross Martin, Pei-Yong Shi, Tomas Cihlar, Danielle P. Porter, Hongmei Mo, Evguenia Maiorova, John P. Bilello

**Affiliations:** a Gilead Sciences, Inc., Foster City, California, USA; b Department of Biochemistry and Molecular Biology, University of Texas Medical Branch, Galveston, Texas, USA

**Keywords:** remdesivir, GS-441524, Nsp12, SARS-CoV-2 variants, COVID-19, antiviral agents

## Abstract

Genetic variation of severe acute respiratory syndrome coronavirus 2 (SARS-CoV-2) has resulted in the emergence and rapid spread of multiple variants throughout the pandemic, of which Omicron is currently the predominant variant circulating worldwide. SARS-CoV-2 variants of concern/variants of interest (VOC/VOI) have evidence of increased viral transmission, disease severity, or decreased effectiveness of vaccines and neutralizing antibodies. Remdesivir (RDV [VEKLURY]) is a nucleoside analog prodrug and the first FDA-approved antiviral treatment of COVID-19. Here, we present a comprehensive antiviral activity assessment of RDV and its parent nucleoside, GS-441524, against 10 current and former SARS-CoV-2 VOC/VOI clinical isolates by nucleoprotein enzyme-linked immunosorbent assay (ELISA) and plaque reduction assay. Delta and Omicron variants remained susceptible to RDV and GS-441524, with 50% effective concentration (EC_50_) values 0.30- to 0.62-fold of those observed against the ancestral WA1 isolate. All other tested variants exhibited EC_50_ values ranging from 0.13- to 2.3-fold of the observed EC_50_ values against WA1. Analysis of nearly 6 million publicly available variant isolate sequences confirmed that Nsp12, the RNA-dependent RNA polymerase (RdRp) target of RDV and GS-441524, is highly conserved across variants, with only 2 prevalent changes (P323L and G671S). Using recombinant viruses, both RDV and GS-441524 retained potency against all viruses containing frequent variant substitutions or their combination. Taken together, these results highlight the conserved nature of SARS-CoV-2 Nsp12 and provide evidence of sustained SARS-CoV-2 antiviral activity of RDV and GS-441524 across the tested variants. The observed pan-variant activity of RDV supports its continued use for the treatment of COVID-19 regardless of the SARS-CoV-2 variant.

## INTRODUCTION

Since the emergence of severe acute respiratory syndrome coronavirus 2 (SARS-CoV-2) in late 2019, two lineages, numerous variants, and subvariants have been detected through genomic surveillance. As with other coronaviruses, SARS-CoV-2 variants emerge through inter- and intramolecular recombination and from heritable errors generated in the viral genome by its error-prone RNA-dependent RNA polymerase (RdRp) (1, 2). Retention of genetic changes in a viral genome may be linked to advantages in replication fitness and/or overcoming selective pressures exerted by the host immune system, an antiviral, or a therapeutic neutralizing antibody. Naturally occurring SARS-CoV-2 variants can be categorized as variants of concern (VOC) or interest (VOI) by the World Health Organization (WHO) or Centers for Disease Control and Prevention (CDC) based on evidence of increased rates of transmission and greater disease severity, detection failures, or potential loss of susceptibility to current vaccines and neutralizing antibodies. The early ancestral A lineage isolates detected in Wuhan, China, and Seattle, WA (WA1 strain), were rapidly replaced worldwide by the B lineage VOC Alpha in 2020 (3). Subsequently, multiple VOI and dominant VOC of the B lineage progressively emerged, with Delta and most recently Omicron replacing prior strains (4–6). The defining genetic changes that differentiate variants predominantly occur in the gene encoding the spike protein, which mediates virus binding, fusion, and entry. However, changes are also detected elsewhere in the viral genomes, resulting in infrequent amino acid substitutions in the Nsp5 3CL main protease (Mpro) and Nsp12 RdRp, the two targets of currently approved SARS-CoV-2 antivirals.

Remdesivir (RDV [VEKLURY]) (7) was the first antiviral approved for the treatment of patients hospitalized with COVID-19, based on evidence that RDV treatment significantly reduced recovery times in clinical trials (8–10). Furthermore, in the PINETREE clinical trial, in which RDV was administered in an outpatient setting, RDV reduced COVID-19-related hospitalization and death by 87% (11). These results led to an expanded FDA approval of RDV for high-risk nonhospitalized individuals with COVID-19 symptoms (12).

RDV is a nucleotide monophosphoramidate prodrug of the parent nucleoside, GS-441524 (13). Following intravenous (i.v.) administration, RDV is metabolized intracellularly to the active triphosphate metabolite (RDV-TP), effectively bypassing the rate-limiting first phosphorylation step of GS-441524. RDV-TP then competes efficiently with cellular ATP for incorporation into the nascent SARS-CoV-2 viral RNA, resulting in cessation of strand synthesis by two separate mechanisms of action (14, 15). Prior to the emergence of SARS-CoV-2, RDV and its parent nucleoside, GS-441524, were shown to inhibit multiple RNA viruses (16–18), including a broad spectrum of coronaviruses, such as SARS-CoV, Middle Eastern respiratory syndrome coronavirus (MERS-CoV), mouse hepatitis virus (MHV), and other zoonotic coronaviruses (19–22). Additionally, potent antiviral activity of RDV was observed in primary lung cells *in vitro* and confirmed *in vivo* across multiple respiratory viruses, including respiratory syncytial virus (RSV) (18), Nipah virus (23), SARS-CoV (20), and MERS-CoV (22).

The RdRp catalytic active site is nearly 100% conserved among coronaviruses; therefore, the observed potency of RDV against other coronaviruses was anticipated to translate to SARS-CoV-2 antiviral activity (21). Both RDV and GS-441524 have demonstrated potency against SARS-CoV-2, with *in vitro* cellular (EC_50_) values ranging from 10 to 120 nM for RDV and 470 to 3,600 nM for GS-441524 (13, 24–27). The *in vivo* efficacy of RDV has been demonstrated in SARS-CoV-2 challenge studies in mice and hamsters (13, 28, 29). Additionally, RDV efficacy was demonstrated in nonhuman primates following several different routes of administration, including i.v., subcutaneous (s.c.), and inhalation (30–32).

The low sequence diversity and high genetic stability of the SARS-CoV-2 RNA replication complex, including the Nsp12 RdRp, observed over time indicate a minimal global risk of preexisting resistance to RDV (33). However, the emergence of each new variant brings a risk of altered susceptibility to vaccine-induced immunity, therapeutic antibodies, or antivirals. In this report, we demonstrate that *in vitro* potencies of RDV and GS-441524 are preserved among the known prominent SARS-CoV-2 variants as well as against recombinant viruses harboring specific substitutions frequently observed in variant Nsp12.

## RESULTS

### Antiviral activity of RDV and GS-441524 against clinical isolates of SARS-CoV-2 variants.

**FIG 1:**
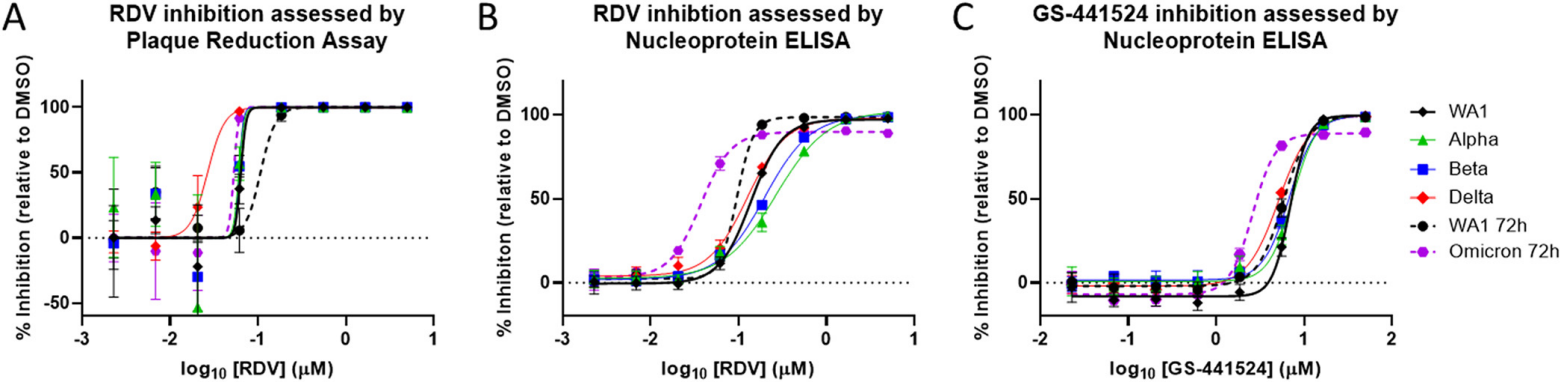
Representative dose-response curves of remdesivir and GS-441524 against SARS-CoV-2 VOC. Shown are dose-response curves of RDV (A and B) and GS-441524 (C) activity against the WA1 reference isolate and SARS-CoV-2 VOCs in A549-ACE2-TMPRSS2 cells by plaque reduction assay (PRA) (A) or ELISA (B and C). In the PRA, infected cell supernatants were harvested at 48 hpi (solid lines) or 72 hpi (dashed lines) and analyzed by plaque assay on Vero-TMPRSS2 cells. For ELISA, infected cells were fixed at ~48 hpi (solid lines) or ~72 hpi (dashed lines) and processed. The data shown are means and standard deviations from representative experiments that were performed in biological quadruplicates (PRA) or triplicates (ELISA) at each compound concentration. Average calculated EC_50_ values and fold change from WA1 reference can be found in Table 1.

*In vitro* RDV antiviral activity was assessed against clinical isolates of an extensive panel of past and present SARS-CoV-2 VOC/VOIs (see Table S1 in the supplemental material). Antiviral activity was initially assessed utilizing a plaque reduction assay (PRA) from culture supernatants harvested at 48 h postinfection (hpi) from A549-ACE2-TMPRSS2 cultures infected with variants at a multiplicity of infection (MOI) of 0.1. The average RDV EC_50_ value for the WA1 reference strain at 48 hpi by PRA was 103 ± 46 nM, while variant EC_50_ values ranged from 13 to 154 nM, representing 0.13- to 1.5-fold changes relative to WA1 (Table 1 and Fig. 1A). Due to low viral yield at 48 hpi, the Omicron variant supernatant was harvested at 72 hpi and compared to a WA1 isolate harvested at the same time. The EC_50_ values at this time point were 141 ± 29 nM and 53 ± 32 nM for the WA1 strain and Omicron variant, respectively. These results indicate that RDV retains potent antiviral activity against all variants evaluated by PRA, including the Delta and Omicron variants, which were both approximately 3-fold more susceptible to RDV than their respective WA1 reference isolates.

**TABLE 1:** Antiviral activity of RDV and GS-441524 against SARS-CoV-2 variants

Variant	RDV plaque reduction assay (*n* = 3–6)		RDV nucleoprotein ELISA (*n* = 5–16)		GS-441524 nucleoprotein ELISA (*n* = 5–12)	
	EC_50_ ± SD (nM)^*a*^	Fold change from WA1^*b*^	Mean EC_50_ ± SD (nM)^*a*^	Mean fold change ± SD from WA1^*c*^	Mean EC_50_ ± SD (nM)^*a*^	Mean fold change ± SD from WA1^*c*^
WA1	103 ± 46	1.0	110 ± 42	1.0	5,600 ± 4,100	1.0
Alpha	94 ± 58	0.92	192 ± 51	1.58 ± 0.48	8,790 ± 6,600	1.22 ± 0.60
Beta	61 ± 9	0.60	141 ± 45	1.19 ± 0.47	7,570 ± 4,400	1.13 ± 0.56
Gamma	154 ± 226	1.5	97 ± 39	0.82 ± 0.42	5,060 ± 2,300	0.79 ± 0.37
Delta	31 ± 13	0.30	70 ± 40	0.59 ± 0.20	3,260 ± 1,300	0.62 ± 0.24
Epsilon	65 ± 32	0.63	210 ± 212	1.94 ± 1.18	4,050 ± 1,700	1.27 ± 0.53
Zeta	87 ± 44	0.85	151 ± 102	1.17 ± 0.40	3,840 ± 1,400	0.93 ± 0.11
Iota	59 ± 28	0.58	258 ± 195^*d*^	2.33 ± 0.74	4,710 ± 1,600	1.43 ± 0.28
Kappa	13 ± 5	0.13	77 ± 50	0.63 ± 0.19	2,100 ± 930	0.53 ± 0.07
Lambda	94 ± 55	0.92	175 ± 138	1.37 ± 0.48	3,890 ± 1,600	0.97 ± 0.10

72 hpi						
WA1	141 ± 29	1.00	95 ± 15	1.0	6,260 ± 1,200	1.0
Omicron	53 ± 32	0.37	42 ± 16^*e*^	0.45 ± 0.13	3,150 ± 1,400^*e*^	0.53 ± 0.28

a Values are the mean ± standard deviation (SD) of the results from independent experiments. *n* represents the number of replicate experiments.

b Fold change calculated from the mean values = (variant mean EC_50_)/(WA1 mean EC_50_).

c A fold change was calculated for each experiment, and a mean fold change ± SD was calculated with these values.

d Statistically significant increase (*P* = 0.015) in EC_50_ value of Iota in the RDV ELISA compared to the WA1 reference isolate at 48 hpi by one-way analysis of variance (ANOVA) with Bonferroni correction for multiple comparisons. All other results for variants at 48 hpi are not statistically different from the matching WA1 reference isolate.

e Statistically significant decrease (*P* ≤ 0.0002) in EC_50_ values of Omicron RDV and GS-441524 ELISA compared to the WA1 reference isolate at 72 hpi by one-way ANOVA with Bonferroni correction for multiple comparisons.

To assess the antiviral effect directly in infected cultures, antiviral testing based on the nucleoprotein enzyme-linked immunoassay (ELISA) was developed and conducted in addition to PRA. The EC_50_ of RDV against the WA1 reference strain by ELISA was 110 ± 42 nM (Table 1 and Fig. 1B), indicating that antiviral potency of RDV is consistent between PRA and ELISA. Variants tested in ELISA had average RDV EC_50_ values ranging from 70 to 258 nM, with observed fold EC_50_ changes from 0.59 to 2.33 (Table 1 and Fig. 1B). Due to the low absorbance signal observed with the Omicron variant at the standard 48-h time point, likely stemming from the reduced *in vitro* replication efficiency of Omicron (34), the assay was also extended to 72 h and compared with WA1 assessed at the same time point. The WA1 reference isolate at 72 hpi had an average RDV EC_50_ of 95 ± 15 nM. Thus, delaying the readout to 72 hpi had no effect on the observed potency of RDV. The Omicron variant was significantly more susceptible to RDV, with an average EC_50_ value of 42 ± 16 nM (*P* ≤ 0.0001), a 0.45-fold change compared to WA1.

The potency of GS-441524, the parent nucleoside of RDV, was also unchanged against all variants and the WA1 reference isolate, as measured by ELISA at 48 hpi. The EC_50_ of GS-441524 at this time point against WA1 was 5,600 ± 4,100 nM, and EC_50_ values ranged from 2,100 to 8,790 nM (0.53- to 1.43-fold change from WA1) against the collection of SARS-CoV-2 variants (Table 1 and Fig. 1C). In agreement with the findings for RDV, GS-441524 was also found to be significantly (*P* = 0.0002) more potent against Omicron than WA1 at 72 hpi, with an EC_50_ value of 3,150 ± 1,400, compared to 6,260 ± 1,200 nM for WA1.

### Nsp12 sequence changes in SARS-CoV-2 variants.

To assess the genetic variation of Nsp12 in the 11 current or previously classified VOC/VOI (Omicron, Delta, Alpha, Beta, Gamma, Epsilon, Zeta, Iota, Kappa, Lambda, and Mu), a total of 5,842,948 SARS-CoV-2 variant sequences from the GISAID (Global Initiative on Sharing Avian Influenza Data) database were evaluated. The highest proportion of analyzed sequences were Delta variants (4,059,836 [69.5%]), followed by Alpha variants (1,158,351 [19.8%]) and Omicron variants (392,056 [6.7%]); the other 8 variants made up the remaining 4.0% (Table S2). We further assessed the genetic variation in spike protein in comparison to Nsp12 across the variants.

The number of amino acid substitutions from WA1 viral isolate sequence in Nsp12 and spike was calculated for each of the 11 variants. Overall, 1 to 6 amino acid substitutions were observed across the different variants, with a frequency of ≥1% of sequences over the 932 amino acid positions in Nsp12, compared to a range of 7 to 45 substitutions over the 1,274 amino acid positions in spike (Fig. S1 and Tables S2 and S3). The most prevalent Nsp12 substitution relative to the consensus ancestral sequence, P323L, was observed with a frequency of >99% and is a lineage-defining Nsp12 substitution for all 11 analyzed variants. The Delta variant contained one additional lineage-defining amino acid change in Nsp12, G671S, which was observed in 97.8% of Delta isolates. No other substitutions were found with a frequency of ≥50% in any of the variants. Analysis of the *nsp7* and *nsp8* genes, which code for RNA binding and processivity cofactors of the replication complex, revealed the proteins coded for by these genes have also been highly conserved throughout the course of the pandemic (Tables S3 and S4). The lineage-defining amino acid change L71F in Nsp7 of the Zeta variant is the only substitution found in >10% of variant isolates. The Nsp7 L71F substitution is distant from the site of RNA replication and is unlikely to impact RDV potency, which is supported by the unchanged susceptibility of Zeta to RDV (Table 1).

Given the recent emergence and high prevalence of the Omicron variant, amino acid substitutions in Nsp12 of Omicron variant were further investigated with a more sensitive frequency cutoff of 0.5%. Among the 6 substitutions (Table 2), P323L and F694Y were the most frequently observed, in 99.5% and 2.0% of Omicron sequences, respectively, while all 4 remaining substitutions had frequencies of ≤1%. In the initial Omicron wave (13 December 2021), F694Y was highly prevalent (41.1% of worldwide isolates and 94.1% of the United Kingdom isolates) in sequences submitted to the GISAID database; however, as the Omicron variant continued to spread, the G694Y substitution rapidly declined in frequency, with only 2.0% of deposited sequences harboring the substitution as of 18 January 2022 (Fig. S2). Interestingly, F694Y is not unique to the Omicron variant as it was also found in 4.9% of Delta variant sequences (Table S2).

**TABLE 2:** Amino acid substitutions with a frequency of ≥0.5% in Nsp12 Omicron sequences

Nsp12 change from WA1 reference isolate	% (*n*) frequency in:	
	Omicron sequences	All available sequences^*a*^
P323L	99.5 (390,020)	98.4 (6,850,250)
F694Y	2.0 (7,822)	3.0 (205,649)
D153Y	0.97 (3,820)	0.10 (6,618)
Q875R	0.86 (3,352)	0.06 (4,286)
G44S	0.59 (2,319)	0.03 (2,336)
I223V	0.54 (2,109)	0.03 (2,333)

a Based on a total of *n* = 7,106,062 sequences from GISAID on 18 January 2022.

Most notably, Nsp12 substitutions previously identified to reduce *in vitro* susceptibility to RDV (19, 35), F480L, V557L, and E802D, were rarely found in our evaluation. Of the 5,842,948 variant sequences evaluated, F480L, V557L, and E802D were only observed in 16 (0.0002%), 24 (0.0004%), and 102 (0.002%) sequences, respectively. Furthermore, only 49 (0.0008%) variant sequences had any alteration at the residue involved in RDV-induced delayed chain termination, S861 (14).

### Structural analysis of Nsp12 substitutions observed in variants.

RDV acts by incorporating its triphosphate metabolite (RDV-TP) into the viral RNA and subsequently causing clashes with the Nsp12 protein at multiple locations, compromising further synthesis (14, 15). Analogs of RDV that produce the same RDV-TP active metabolite, such as GS-441524, exert their inhibitory activity via the same mechanism of action. At present, a structure of the preincorporated state of RDV-TP in the RdRp active site is still unavailable. We built a model, described previously (14, 15, 36), based on an existing structure of the polymerase complex, Nsp12/(Nsp8)_2_/Nsp7/(Nsp13)_2_, with primer and template RNA (PDB no. 6XEZ) (37). Using this model, we assessed the potential impact of each Nsp12 amino acid substitution identified in the analyzed variants on the affinity of RDV-TP for the RdRp active site.

**FIG 2:**
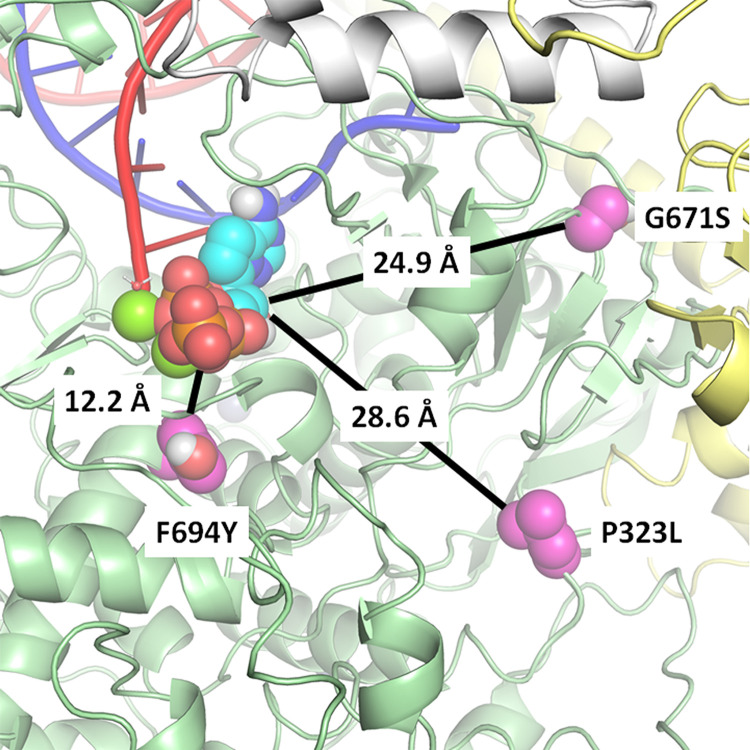
Structural model of Nsp12 highlighting key amino acid substitutions in relation to the active site. Preincorporated remdesivir triphosphate (RDV-TP) was modeled into the cryo-EM structure of the polymerase complex (PDB no. 6XEZ) (37). Nsp12 is represented in green, with Nsp8 in yellow, and Nsp7 in white. The prevalent amino acid substitution P323L, seen in all variants, was measured to be 28.6 Å from RDV-TP (P323 Cα-RDV-TP C1′), whereas G671S, seen in the Delta variant, is at 24.9 Å. Of all the amino acid substitutions reported here, F694Y, seen at low frequency in Delta and Omicron, comes closest to the active site, at 12.2 Å. A computational analysis suggests that the substitutions have no meaningful impact on RDV-TP binding affinity.

As seen in Fig. 2, the two most common amino acid substitutions, P323L, seen in all variants, and G671S, observed in Delta, are 28.6 Å and 24.9 Å, respectively, from the preincorporated RDV-TP (measured from the amino acid Cα to RDV-TP’s C1′). Of all the low-frequency substitutions identified, only F694Y, found in 2 to 5% of Omicron and Delta isolates, is in close proximity to the RdRp active site. Measured to be 12.2 Å from the RDV-TP, the residue is not in direct contact with the inhibitor but is close enough to have an indirect conformational effect. However, an evaluation of its impact on RDV-TP binding affinity using a molecular mechanics generalized Born surface area (MM-GBSA) approach resulted in no meaningful difference, likely because of the relatively conservative change from phenylalanine to tyrosine (38).

Most low-frequency amino acid substitutions in Omicron and other variants occur on the surface of Nsp12, away from the polymerase active site (Fig. S3 and S4). While the dynamics of incorporation and RDV-TP inhibition are complex events, this structural analysis suggests little reason to expect a significant impact on the efficacy of RDV and GS-441524.

### Potency against recombinant SARS-CoV-2 expressing the prevalent variant amino acid substitutions.

**FIG 3:**
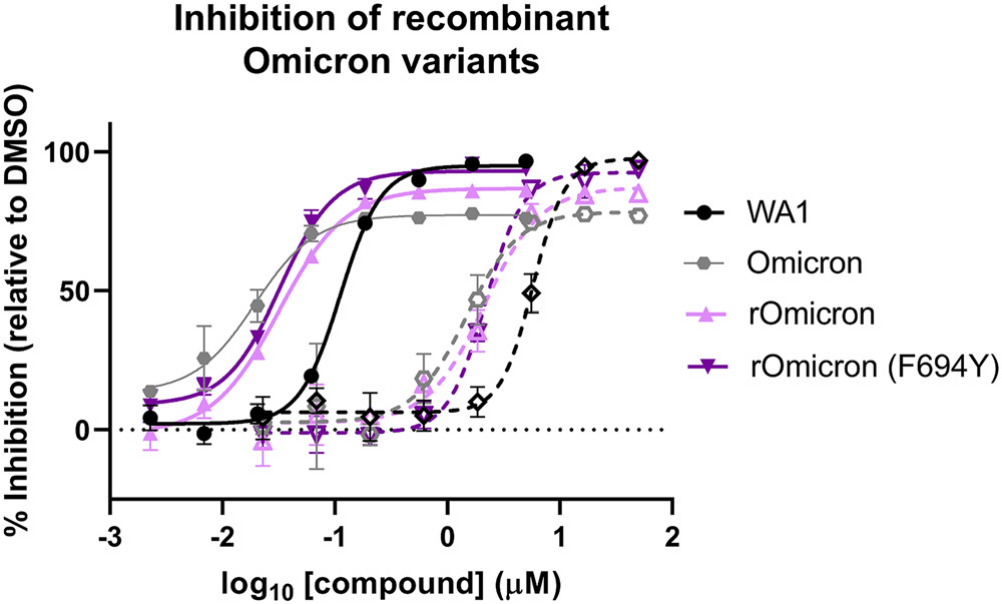
Recombinant Omicron viruses retain susceptibility to RDV and GS-441524. Shown are ELISA dose-response curves of RDV (solid lines and solid symbols) and GS-441524 (dashed lines and open symbols) activity against recombinant Omicron viruses with (dark purple) or without (light purple) the F694Y substitution compared with WA1 (black), and Omicron (gray) clinical isolates run in parallel. The data shown are means and standard deviations from a representative 72-hpi nucleoprotein ELISA that was performed with biological triplicates at each compound concentration. Average calculated EC_50_ values are in Table 3.

The Omicron clinical isolate evaluated did not contain the Nsp12 F694Y substitution found at high frequency in early U.K. Omicron isolates (Fig. S2). Due to the initial prevalence in Omicron variants and proximity to the RdRp active site, we sought to assess RDV and GS-441524 activity against recombinant Omicron (rOmicron) viruses with and without the Nsp12 F694Y substitution. By ELISA, the RDV EC_50_ values were 44 ± 9 nM and 31 ± 5 nM (Table 3 and Fig. 3) against rOmicron and rOmicron F694Y, respectively. Similarly, potency was preserved for GS-441524 against both recombinant viruses, with EC_50_ values of 2,440 ± 100 nM (rOmicron) and 1,900 ± 400 nM (rOmicron F694Y). RDV and GS-441524 were similarly potent against the two recombinant Omicron viruses and an Omicron clinical isolate run in parallel, with all three viruses showing increased susceptibility to both RDV and GS-441524 compared to the WA1 isolate by ELISA (Table 3 and Fig. 3).

**TABLE 3:** RDV and GS-441524 potencies against recombinant Omicron SARS-CoV-2 viruses

Virus	Mean EC_50_ ± SD (nM)^*a*^	
	RDV (*n* = 3–4)	GS-441524 (*n* = 3–4)
WA1	94 ± 19	5,490 ± 800
Omicron	51 ± 22^*b*^	3,510 ± 1,800^*b*^
rOmicron	44 ± 9^*b*^	2,440 ± 100^*b*^
rOmicron F694Y	31 ± 5^*b*^	1,900 ± 400^*b*^

a Values are the mean ± SD of the results from independent experiments. *n* represents the number of replicate experiments.

b Statistically significant decrease (*P* ≤ 0.001) in EC_50_ value of Omicron and rOmicron viruses from the WA1 reference at 72 hpi by one-way ANOVA with Bonferroni correction for multiple comparisons. No statistical differences were observed between any of the Omicron and rOmicron viruses.

**FIG 4:**
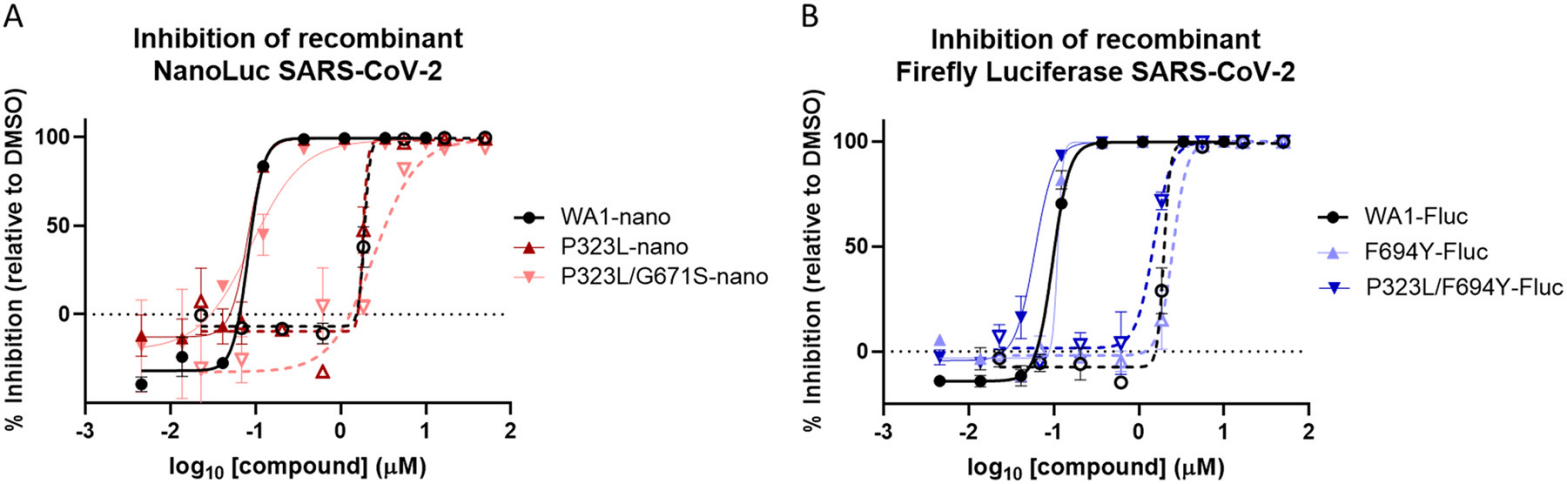
Prevalent Nsp12 substitutions in Delta and Omicron retain susceptibility to RDV and GS-441524. Shown are dose-response curves of RDV (solid lines and solid symbols) and GS-441524 (dashed lines and open symbols) activity against recombinant viruses with/without prevalent Nsp12 substitutions containing a Nano luciferase (Nluc) (A) or firefly luciferase (Fluc) (B) transgene. The data shown are means and standard deviations from a representative experiment that was performed in biological duplicates at each compound concentration. Average calculated EC_50_ values and fold change from recombinant WA1 references are in Table 4.

We next sought to evaluate the *in vitro* potency of RDV and GS-441524 against other Nsp12 amino acid substitutions alone or in combination that were identified at high frequency (>15%) in any specific variant. Recombinant SARS-CoV-2 WA1 viruses containing a Nano luciferase (Nluc) transgene, and the wild-type or mutated *nsp12* sequences were rescued and tested for RDV and GS-441524 susceptibility. Monitoring Nluc signal from infected cells at 48 hpi, we observed an RDV EC_50_ value of 80 ± 21 nM for WA1 recombinant virus, while viruses containing either the P323L substitution alone or the P323L/G671S double substitution found in the Delta variant had RDV EC_50_ values of 71 ± 26 nM and 104 ± 20 nM, respectively, resulting in a 1.2-fold change or less relative to WA1 (Table 4 and Fig. 4A). Modification of *nsp12* with a sequence encoding G671S alone failed to rescue infectious virus after several independent attempts, a finding that complements prior evidence suggesting Nsp12 P323L conveys a growth advantage (39). Recombinant viruses containing either P323L, F694Y, or the P323L/F694Y double substitution in a WA1 firefly luciferase (Fluc) recombinant virus background were similarly susceptible to RDV (EC_50_ values within 1.2-fold of WA1) (Table 4 and Fig. 4B). GS-441524 antiviral potency was also maintained against recombinant SARS-CoV-2 viruses harboring the Nsp12 P323L, P323L/G671S, and P323L/F694Y substitutions, with fold changes of 0.73 to 1.83 relative to WA1 (Table 4). Collectively, these data confirm that antiviral potencies of RDV and GS-441524 remain unchanged against viruses harboring the prevalent Nsp12 substitutions currently identified in isolates of SARS-CoV-2 variants.

**TABLE 4:** RDV and GS-441524 potencies against recombinant SARS-CoV-2 harboring prevalent Nsp12 substitutions

Recombinant virus	RDV (*n* = 2–6)		GS-441524 (*n* = 2)	
	Mean EC_50_ ± SD (nM)^*a*^	Mean fold change ± SD from WA1^*b*^	Mean EC_50_ ± SD (nM)^*a*^	Mean fold change ± SD from WA1^*b*^
WA1-Nluc	80 ± 21	1.0	1,880 ± 40	1.0
P323L	71 ± 26	0.95 ± 0.42	1,580 ± 370	0.84 ± 0.21
P323L/G671S	104 ± 20	1.22 ± 0.31	3,450 ± 1,400	1.83 ± 0.71

WA1-Fluc	99 ± 2	1.0	2,230 ± 380	1.0
F694Y	112 ± 2	1.14 ± 0.01	2,250 ± 380	1.04 ± 0.34
P323L/F694Y	63 ± 4	0.63 ± 0.03	1,620 ± 180	0.73 ± 0.04

a Values are the mean ± SD of the results from independent experiments. *n* represents the number of replicate experiments.

b A fold change was calculated for each experiment, and the mean fold change ± SD was calculated with these values.

## DISCUSSION

Over the past 2 years of the SARS-CoV-2 pandemic, the rapid evolution of the virus has led to the emergence of multiple viral variants. Since the beginning of pandemic, the WHO declared 5 of these variants as VOC that could be associated with more severe disease and/or increased rate of transmission. While the SARS-CoV-2 antiviral activity of RDV has previously been well characterized both *in vitro* and *in vivo* (13, 24, 26, 29–31), most studies have been conducted using the ancestral WA1 isolate. Here, we sought to fully characterize the antiviral potency of RDV and its parent nucleoside, GS-441524, against a panel of the most significant SARS-CoV-2 variants, including all the major VOC. Utilizing PRAs and ELISAs in parallel, we observed a general agreement in potency between assays, with most RDV EC_50_ values observed near 100 nM, indicating nucleoprotein levels correlated with released infectious virus. Findings from both assays revealed all variants to have RDV EC_50_ values within 2.4-fold of those of WA1. The Iota variant was the only variant with >2-fold change in potency for RDV compared with WA1 by ELISA. However, the EC_50_ values against Iota observed by PRA and for GS-441524 by ELISA were similar to those of WA1, indicating that the Iota variant remains susceptible to RDV and GS-441524. Importantly, both Delta and Omicron variants, the two most recent strains in predominant circulation with increased severity and elevated transmission, respectively, are highly susceptible to both RDV and GS-441524. Interestingly, Omicron is significantly more susceptible to RDV and GS-441524, although the reasons for this are not understood, as there are no substitutions in Nsp12 that would predict increased potency. This is in line with several other SARS-CoV-2 inhibitors targeting the SARS-CoV-2 RdRp or main protease with different mechanisms of action, which also exhibit increased potency against Omicron compared to ancestral strains (40–42).

The potency observed for the GS-441524 parent nucleoside against all variants was 20 to 75 times lower than that for RDV, consistent with previous findings in A549 cells (13). As observed with RDV, GS-441524 maintained potency against all clinical isolates of variants tested, with a maximum fold change of 1.4 compared with WA1. Although the active triphosphates for GS-441524 and RDV are identical, it was important to confirm pan-variant GS-441524 potency because orally bioavailable prodrug options for delivery of GS-441524 are under exploration (43, 44).

The sequence analyses presented here and by others (22, 45) have found the *nsp12* gene, encoding the RNA-dependent RNA polymerase, of variants to be remarkably stable over the last 2 years. Only two substitutions, P323L (among all variants) and G671S (in the Delta variant), have an observed prevalence of >15% among sequenced variant isolates (Table S2). In contrast, multiple substitutions in the spike protein have been observed in all variants (Fig. S1 and Table S3), which can result in immune evasion and reduced efficacy of monoclonal antibodies (46, 47). Structural analysis of P323L, G671S, and F694Y (a highly prevalent substitution in early Omicron isolates) found each of these substitutions to be unlikely to reduce susceptibility to RDV. We confirmed RDV and GS-441524 retained antiviral activity against recombinant viruses containing each of these substitutions individually or in various combinations. The findings were consistent with antiviral assessments performed in clinically isolated variants containing these Nsp12 substitutions, in which RDV and GS-441524 have similar potencies to WA1. Therefore, future variants containing P323L, G671S, or F694Y individually or in combination are likely to remain susceptible to RDV and GS-441524.

Nsp12 mutations selected through *in vitro* passaging that are known to confer RDV resistance in coronaviruses (19, 35) are noticeably lacking from the sequence analysis of clinical samples. These mutations were observed in <0.001% of sequences analyzed, indicating that despite the widely prevalent use of RDV to treat COVID-19 in >10 million hospitalized patients over the course of the pandemic, emergence of RDV-resistant viruses is rare (45). The low prevalence of RDV resistance mutations in clinical SARS-CoV-2 samples may in part be due to a reduced fitness of viruses harboring these mutations, as some RDV resistant mutations have been found to have delayed replication kinetics (48). However, with the recent expansion of RDV indication to treat COVID-19 earlier in the course of viral infection through outpatient use (11, 12) or potential future use of orally bioavailable prodrugs of GS-441524, a sustained surveillance for emergence of resistance will need to continue.

In summary, we confirmed with several assay systems that past and present SARS-CoV-2 VOC and VOI retain *in vitro* susceptibility to both RDV and its parent nucleoside, GS-441524. These findings highlight that both RDV and GS-441524 exhibit pan-variant SARS-CoV-2 activity and support the continued clinical use of RDV in approved patient populations.

## MATERIALS AND METHODS

### Reagents.

Remdesivir (RDV) and GS-441524 were synthesized at Gilead Sciences, Inc. Validation of chemical identities was determined by nuclear magnetic resonance (NMR) and liquid chromatography mass spectrometry (LC-MS), and purity of >95% was assessed by high-performance liquid chromatography (HPLC) (7, 18). Compounds were solubilized in 100% dimethyl sulfoxide (DMSO) at a concentration of 10 mM.

### Viruses and cells.

Vero-TMPRSS2 cells expressing human transmembrane serine protease 2 (hTMPRSS2) (49) were purchased from JCRB Cell Bank (catalog no. JCRB 1818), National Institutes of Biomedical Innovation, Health and Nutrition. A549-hACE2 cells that stably express human angiotensin-converting enzyme 2 (hACE2) were established and provided by the University of Texas Medical Branch (50). A549-ACE2-TMPRSS2 cells (catalog no. a549-hace2tpsa) were purchased from InvivoGen (San Diego, CA). All cells were maintained at 37°C and 5% CO_2_ in Dulbecco’s minimum essential medium (DMEM) with GlutaMAX (Gibco catalog no. 10569-010) supplemented with 10% heat-inactivated fetal bovine serum (FBS) (HyClone catalog no. SH30396.03), 100 U/mL penicillin, 100 μg/mL streptomycin (Gibco catalog no. 15140-122), and the appropriate selection agents—1 mg/mL Geneticin (Vero-TMPRSS2), 10 μg/mL blasticidin (A549-hACE2), or 0.5 μg/mL puromycin and 100 μg/mL hygromycin B (A549-ACE2-TMPRSS2). All cells were passaged 2 to 3 times per week with 0.25% trypsin–0.02% EDTA (Gibco catalog no. 25200056). Cells used in all experimental set-ups were between passage 5 (P5) and P30.

SARS-CoV-2 isolates (Table S1) were acquired through the World Reference Center for Emerging Viruses and Arboviruses at the University of Texas Medical Branch (Delta and Epsilon) and BEI Resources, National Institute of Allergy and Infectious Diseases (NIAID), National Institutes of Health (NIH). Isolates obtained from BEI Resources were deposited by the CDC (WA1 reference and Lambda), Bassam Hallis (Alpha), Alex Sigal and Tulio de Oliveira (Beta), the National Institute of Infectious Diseases (Gamma), Andrew S. Pekosz (Omicron and Zeta), Mehul Suthar and Benjamin Pinsky (Kappa), and David D. Ho (Iota).

All viruses were propagated 1 to 2 times in Vero-TMPRSS2 cells as follows. A total of 1 × 10^7^ Vero-TMPRSS2 cells were seeded into a T225 flask in Vero-TMPRSS2 maintenance medium and incubated overnight at 37°C and 5% CO_2_. The following day, the medium was aspirated and replaced with 25 mL of DMEM supplemented with 2% FBS (infection medium) and infected with 10 μL of passage 0 (P0) stocks. The flasks were returned to 37°C and 5% CO_2_ until only 10 to 20% of viable cells remained (typically 36 to 72 hpi). The supernatant was harvested into a 50-mL Falcon tube and centrifuged at 2,000 × *g* for 5 min to pellet cellular debris. The clarified supernatant was then transferred to a clean Falcon tube, aliquoted as a working P1 stock into 100- to 250-μL aliquots, and frozen at −80°C. The titer of the P1 stock was determined by plaque formation assay (PFA). If a second passage was required, the procedure described above was repeated using the P1 stock to inoculate.

### Plaque formation assay.

A total of 3 × 10^5^ Vero-TMPRSS2 cells/well were seeded into 12-well plates in 1 mL of maintenance medium and incubated overnight at 37°C and 5% CO_2_. The following day, cell confluence was confirmed to be >95% by visualization under a light microscope. Samples for analysis were serially diluted 10-fold in infection medium (DMEM plus 2% FBS) up to a final dilution of 10^−5^ or 10^−6^. Spent supernatant was aspirated and replaced with 100 μL of serially diluted inoculum/well, and culture plates were returned to the incubator for 1 h with gentle rocking every 15 min. Following incubation, 2 mL of prewarmed overlay medium (DMEM with 2% FBS, 1× penicillin-streptomycin, and 1.5% carboxymethylcellulose) was added to each well. Cells were then incubated without agitation for 3 days or 4 days (only for Omicron variants), at which point, 2 mL of crystal violet fix/stain solution was added to each well. Cells were incubated at room temperature overnight. Supernatants containing the crystal violet solution were discarded, and wells were washed with water 2 to 4 times each until plaques were visible and washes were clear of crystal violet residue. Plaques were counted manually from the most dilute wells consistently containing >5 PFU.

### Plaque reduction assay.

A total of 5 × 10^4^ A549-ACE2-TMPRSS2 cells were suspended into 500 μL maintenance medium and seeded into each well of a 48-well plate (Corning). Plates were incubated at 37°C with 5% CO_2_ overnight, after which the medium was aspirated and 250 μL of infection medium (DMEM plus 2% FBS) was added to each well. Serial 3-fold dilutions of RDV in DMSO were added to each well using a Tecan D300e digital liquid dispenser. The DMSO concentrations were normalized to that of the highest compound concentration (DMSO at less than <0.1% in the final solution). SARS-CoV-2 was diluted into infection medium to 1 × 10^5^ PFU/mL, and 50 μL of inoculum was added to each well to result in a multiplicity of infection (MOI) of 0.1. At 48 or 72 hpi (for Omicron and the WA1 reference), the supernatant was transferred to a clean 48-well plate, and the plate was sealed and frozen at −80°C until ready for analysis using the PFA as described above. PFU counts for each variant were normalized to the DMSO controls for each variant (DMSO average = 0% inhibition). Due to the cumbersome nature of the PRA, all variants could not be read out simultaneously; therefore, fold change calculations for this assay were assessed by taking the average EC_50_ for each variant divided by the average EC_50_ of the WA1 reference.

### Nucleoprotein ELISA.

A total of 3 × 10^4^ A549-ACE2-TMPRSS2 cells in 100 μL DMEM (supplemented with 10% FBS and 1× penicillin-streptomycin) were seeded into each well of a 96-well plate and incubated overnight. The following day, medium was aspirated, and 100 μL of DMEM containing 2% FBS was added to each well. Three-fold serial dilutions of RDV or GS-441524 (in triplicate) were added to each well using an HP D300e digital dispenser with a final volume of 200 μL/well. Immediately after compound addition, cells were infected with 1.5 × 10^3^ PFU of the relevant SARS-CoV-2 variant diluted in 100 μL of DMEM supplemented with 2% FBS, resulting in an MOI of 0.05. Plates were centrifuged for 1 min at 500 × *g* and then incubated at 37°C with 5% CO_2_ for 2 days (or 3 days for Omicron strains and the WA1 reference), after which medium was aspirated and cells were fixed with 100% methanol for 10 min at room temperature (RT). The methanol was removed, and plates were air dried for 10 min at RT, followed by 1 h of incubation with 100 μL/well of blocking buffer (phosphate-buffered saline [PBS] with 10% FBS, 5% nonfat dry milk, and 0.1% Tween 20) for 1 h at 37°C. The blocking buffer was then aspirated, and 50 μL of a 1:4,000 dilution of rabbit anti-SARS-CoV-2 nucleocapsid (NC) antibody (MA536086; Invitrogen) in blocking buffer was added and incubated for 2 h at 37°C. Plates were washed 4 times with 200 μL/well of PBS containing 0.1% Tween 20 prior to addition of 50 μL/well of horseradish peroxidase (HRP)-conjugated goat anti-rabbit IgG (GtxRb-003-FHRPX; ImmunoReagents) diluted 1:4,000 in blocking buffer. Plates were again incubated for 1 h at 37°C and then washed 4 times with 200 μL PBS with 0.1% Tween 20. One hundred microliters 3,3′,5,5′-tetramethylbenzidene (TMB) reagent (ENN301; Thermo Scientific) was added to each well and allowed to incubate at RT until visible staining of the positive-control wells—usually 5 to 10 min. The reaction was stopped with addition of 100 μL/well of TMB stop solution (5150-0021; SeraCare). The absorbance was then read at 450 nm using an EnVision plate reader. Fold change for variants was calculated for each experiment, with comparison to the relevant WA1 reference. Fold change across all experiments was then averaged to obtain the final reported values.

### SARS-CoV-2 sequence analysis.

The tabulated amino acid substitutions from the WA1 reference isolate (MN985325) for a total of 5,842,948 SARS-CoV-2 genome sequences were obtained from GISAID EpiCov database as of 18 January 2022 (https://www.gisaid.org/) (51). Sequences with a length of <29,000 nucleotides or that contained >5% of ambiguous bases across the genome were excluded from analyses. The sequences were further categorized into 11 VOC/VOI according to the PANGO lineage using Pangolin software (52). The Regeneron COVID-19 Dashboard web portal (https://covid19dashboard.regeneron.com) was used to assess the overall prevalence of mutations in 7,106,062 unfiltered sequences from the GISAID database on 18 January 2022. Lineage-associated amino acid changes were obtained from PANGO lineage web portal (https://cov-lineages.org/).

### Protein structure modeling and visualization.

The model of preincorporated RDV-TP in the active site of the SARS-CoV-2 polymerase complex was developed from the nucleoside triphosphate (NTP)-free cryo-electron microscopy (cryo-EM) structure 6XEZ and has been described elsewhere (37). The variant mutations P323L, P323L/G671S, and P323L/F694Y were introduced and optimized by conducting a side-chain rotamer optimization and minimization of the mutated residues and surrounding residues within 5 Å using Prime. The impact of each mutation on the predicted binding affinity to RDV-TP was assessed with an MM-GBSA residue scan within Bioluminate.

### Site-directed mutagenesis and recombinant virus rescue.

To produce recombinant SARS-CoV-2 virus, we utilized a SARS-CoV-2 reverse-genetics system as previously described (24, 53), which was slightly modified by fusing plasmids F1 to F3 into a single plasmid, making it a 3-plasmid reverse-genetics system producing infectious virus containing either Nano luciferase (Nluc) or the firefly luciferase (Fluc) transgene. Desired substitutions in *nsp12* of the SARS-CoV-2 genome were added to the *nsp12*-containing F4 plasmid using the Quick-Change PCR protocol with Platinum SuperFI II PCR master mix (Thermo Fisher Scientific catalog no. 12361010) following the manufacturer’s protocols. The primers used to engineer specific mutations were SARS_CoV2_NSP12_P323L_Fw (5′-GTTCCCAC**T**TACAAGTTTTG-3′) and SARS_CoV2_NSP12_P323L_Rv (5′-CAAAACTTGTA**A**GTGGGAAC-3′) for P323L SARS_CoV2_NSP12_F694Y_Fw (5′-GCTAATAGTGTTT**A**TAACATTTGTC-3′) and SARS_CoV2_NSP12_F694Y_Rv (5′-GACAAATGTTA**T**AAACACTATTAGC-3′) for F694Y, and SARS_CoV2_NSP12_G671S_Fw (5′-GTCATGTGTGGC**A**GTTCACTATATG-3′) and SARS_CoV2_NSP12_G671S_Rv (5′-CATATAGTGAAC**T**GCCACACATGAC-3′) for G671S. Substitutions (highlighted in boldface in primer sequences) were sequence confirmed, and then validated plasmids were digested with either BsaI or Esp3I. Cut plasmids were then ligated together using T4 DNA ligase, and the ligated product was *in vitro* transcribed into RNA. The RNA products were then electroporated into Vero-TMPRSS2 cells and monitored until extensive cytopathic effect was observed and P0 virus harvested. Titers of P0 virus stocks were determined, and virus was passaged to P1 as described above for propagation of clinical isolates. Virus used for experiments was either P1 (Fluc) or P2 (Nluc).

### Construction of a recombinant Omicron SARS-CoV-2.

Recombinant Omicron SARS-CoV-2 was constructed by engineering the complete mutations from Omicron variant (GISAID EPI_ISL_6640916) into an infectious cDNA clone of clinical isolate USA-WA1/2020 (54). All mutations were introduced into the infectious cDNA clone of USA-WA1/2020 using PCR-based mutagenesis as previously described (55). An additional recombinant Omicron SARS-CoV-2 was generated bearing the F694Y substitution in Nsp12 by the methods detailed above. rOmicron viruses were analyzed using the nucleoprotein ELISA following the protocol used for clinical isolates.

### Antiviral activity assessment from recombinant luciferase containing viruses.

For Nluc readouts, 1.2 × 10^4^ A549-hACE2 cells per well were suspended in 50 μL infection medium, seeded into a white clear-bottom 96-well plate (Corning), and incubated overnight at 37°C with 5% CO_2_. On the following day, compounds were added directly to cultures as 3-fold serial dilutions with a Tecan D300e digital liquid dispenser, with DMSO volumes normalized to that of the highest compound concentration (final DMSO concentration of <0.1%). SARS-CoV-2-Nluc viruses were diluted to an MOI of 0.05 and aliquoted at 50 μL/well. At 48 hpi, 75 μL Nluc substrate solution (Promega) was added to each well. Luciferase signals were measured using an Envision microplate reader (Perkin Elmer).

For firefly luciferase readouts, the assay setup was the same as for the Nluc assay, except cells were infected with SARS-CoV-2-Fluc viruses at an MOI of 1.0, and at 48 hpi, 100 μL One-Glo luciferase substrate solution (Promega) was added to each well prior to reading the signal on the Envision plate reader (Perkin Elmer).

### EC_50_ determinations.

The half-maximal effective concentration (EC_50_) is defined as the compound concentration at which there was a 50% reduction in plaque formation (PRA), luciferase signal, or nucleoprotein expression (ELISA) relative to infected cells with DMSO alone (0% inhibition) and uninfected control cells (100% inhibition). EC_50_ values were determined using GraphPad Prism 8.1.2 with nonlinear regression curve fits. Constraints were used when required to ensure the bottom or top of the fit curves were close to 0 and 100, respectively.
