# Supplementary material for: Remdesivir and GS-441524 Retain Antiviral Activity against Delta, Omicron, and Other Emergent SARS-CoV-2 Variants
Source: Antimicrob Agents Chemother. 2022 May 9;66(6):e00222-22. doi: 10.1128/aac.00222-22 (PMC9211395; doi:10.1128/aac.00222-22)
Supplement: Supplemental file 1 — Fig. S1 to S4 and Tables S1 to S5. Download aac.00222-22-s0001.pdf, PDF file, 0.6 MB [file aac.00222-22-s0001.pdf]

Supplemental Materials

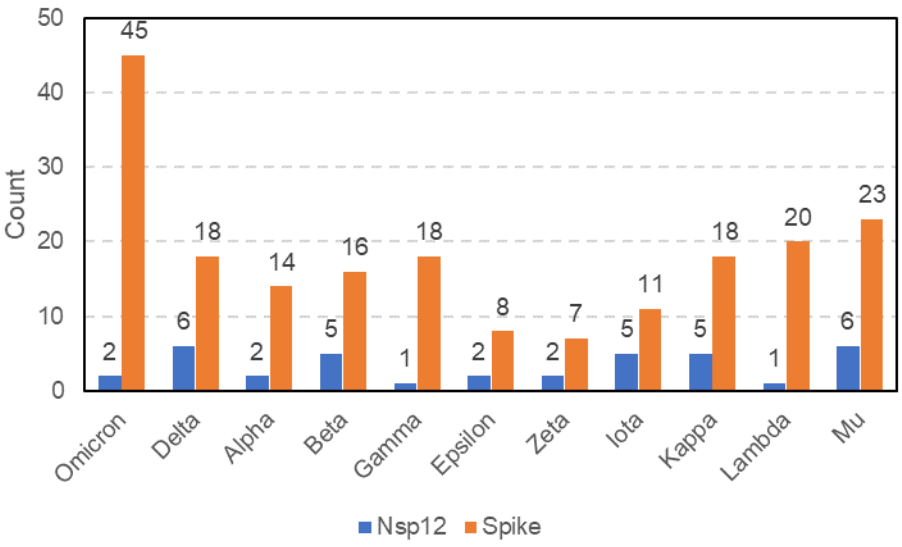

Supplemental Figure 1. Amino acid substitutions in Nsp12 and Spike. The total number of amino acid substitutions in Nsp12 compared to Spike from each variant of concern or variant of interest. Actual substitutions for each variant are found in Supplemental Tables 1 (Nsp12) and 2 (Spike).

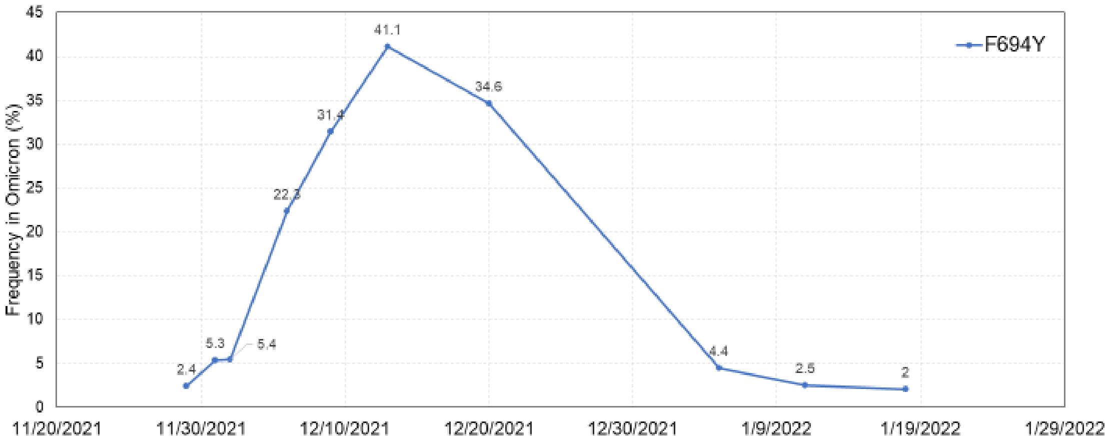

Date	11/29/21	12/1/21	12/2/21	12/6/21	12/9/21	12/13/21	12/20/21	1/5/22	1/11/22	1/18/22
% in Omicron isolates (Total isolates N)	2.4 (165)	5.3 (279)	5.4 (298)	22.3 (542)	31.4 (1,127)	41.1 (2,840)	34.6 (13,659)	4.4 (116,830)	2.5 (248,913)	2.0 (392,056)
% UK isolates with 694Y (Total UK Isolates N)	100.0 (4)	100.0 (15)	100.0 (15)	95.2 (126)	93.7 (378)	94.1 (1,240)	45.5 (8,766)	6.2 (66,280)	4.1 (118,013)	2.9 (172,297)

Supplemental Figure 2. F694Y frequency in Omicron variant over time. Omicron sequences were obtained from GISAID at ten different timepoints. Frequency of F694Y in all omicron sequences at each timepoint are plotted. The frequency among UK isolates is shown in the data table below the plot.

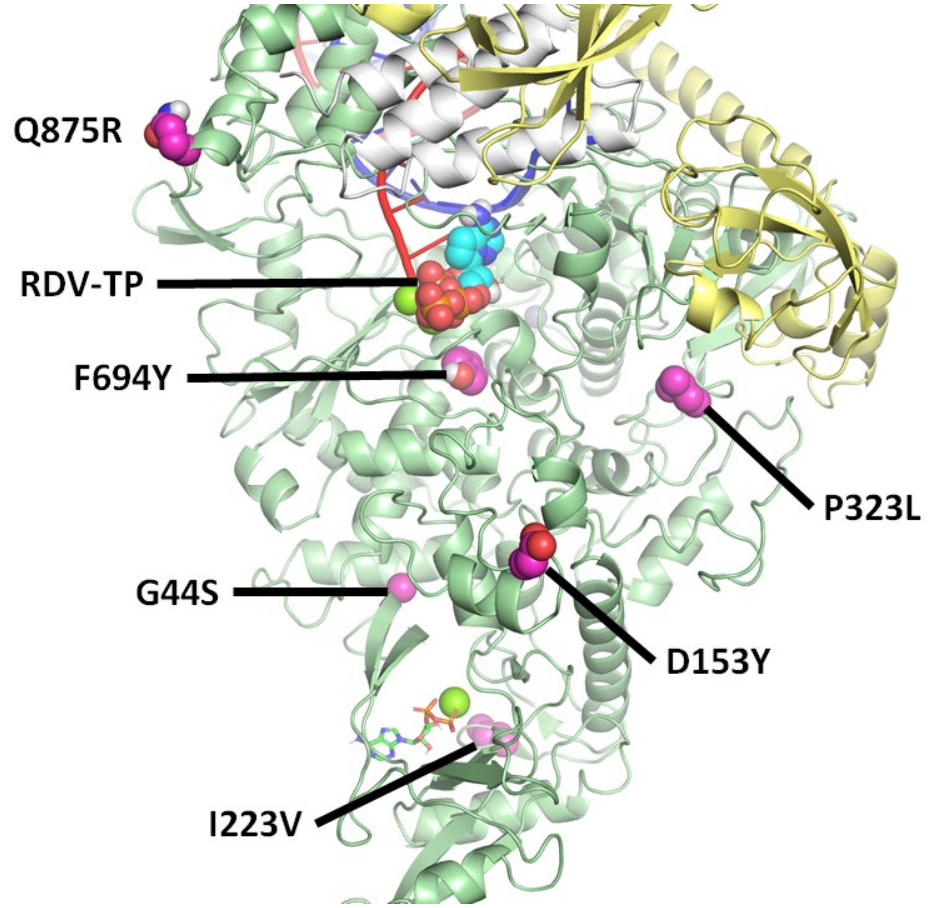

Supplemental Figure 3. Location of Omicron Nsp12 amino acid substitutions with frequency >0.5%. Amino acid substitutions identified in Table 2 are mapped onto the model of the polymerase complex with pre-incorporated RDV-TP. All substitutions occur on the surface of Nsp12, away from the active site, apart from F694Y. Nsp12 is shown in green, Nsp8 in yellow, Nsp7 in white, template RNA in blue, and primer RNA in red.

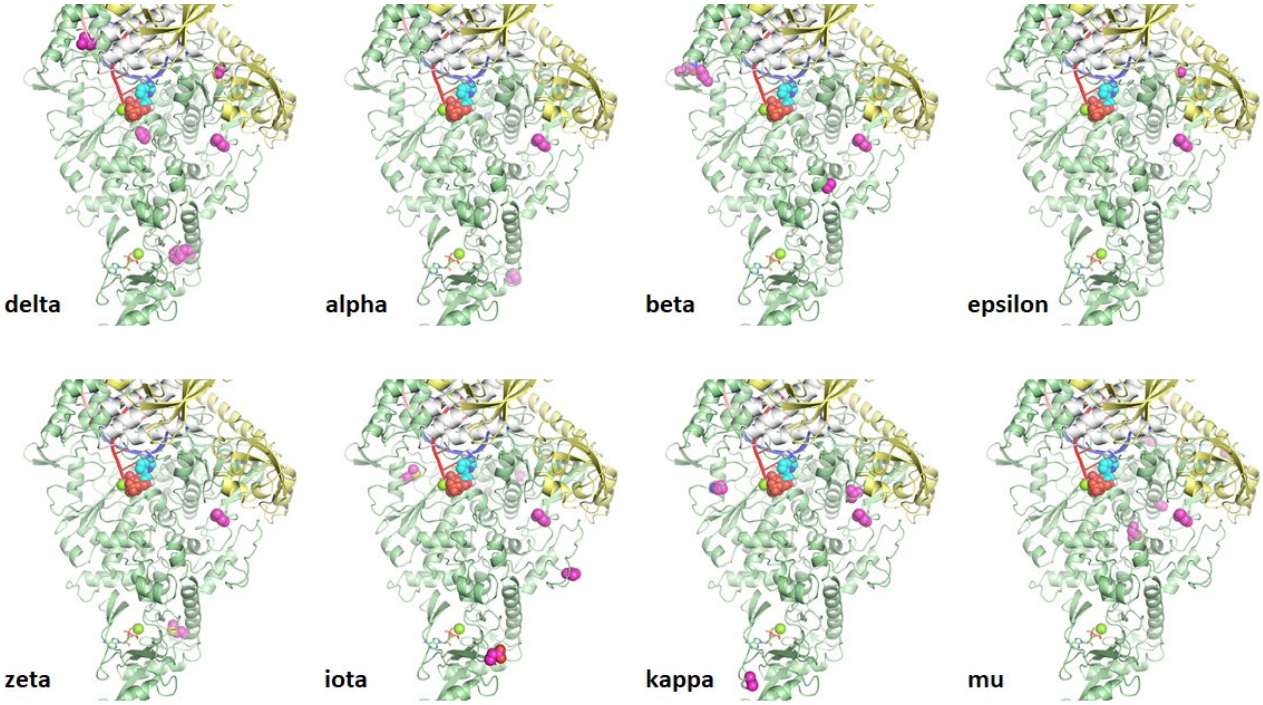

Supplemental Figure 4. Location of variant Nsp12 amino acid substitutions with frequency >1%. Amino acid substitutions identified in Supplemental Table 1 are mapped onto the model of the polymerase complex with pre-incorporated RDV-TP. Gamma and Lambda are not shown, since substitutions are limited to P323L, while Omicron substitutions are shown in Supplemental Fig. 3. With the exception of F694Y, seen in Delta, the substitutions are all remote from the polymerase active site. Nsp12 is shown in green, Nsp8 in yellow, Nsp7 in white, template RNA in blue, and primer RNA in red. Amino acid substitutions are shown in magenta.

Supplemental Table 1. SARS-CoV-2 variant clinical isolates and sources

Variant	Isolate	Lineage	Source (Cat #)	GISAID ID	Depositor
	hCoV-19/USA-WA1/2020 (WA1)	A	BEI (NR-52281)	EPI_ISL_404895	Center for Disease Control and Prevention
Alpha	hCoV-19/England/204820464/2020	B.1.1.7	BEI (NR-54000)	EPI_ISL_683466	Bassam Hallis
Beta	hCoV-19/South Africa/KRISP-K005325/2020	B.1.351	BEI (NR-54009)	EPI_ISL_678615	Alex Sigal and Tulio de Oliveira
Gamma	hCoV-19/Japan/TY7-503/2021	P.1	BEI (NR-54982)	EPI_ISL_877769	National Institutes of Infectious Disease
Delta	GNL-1205	B.1.617.2	Source: UTMB		
Epsilon	GNL-354	B.1.429	Source: UTMB		
Zeta	hCoV-19/USA/CA/VRLC012/2021	P.2	BEI (NR-55439)	EPI_ISL_1364506	Andrew S. Pekosz
Iota	hCoV-19/USA/NY-NP-DOH1/2021	B.1.526	BEI (NR-55359)	EPI_ISL_1080761	David D. Ho (Columbia University)
Kappa	hCoV-19/USA/CA-Stanford-15_S02/2021	B.1.617.1	BEI (NR-55486)	EPI_ISL_1675223	Dr Mehul Suthar and Dr. Benjamin Pinsky
Lambda	hCoV-19/Peru/un-CDC-2-4069945/2021	C.37	BEI (NR-55654)	EPI_ISL_1591097	Center for Disease Control and Prevention
Omicron	hCoV-19/USA/MD-HP20874/2021	B.1.1.529	BEI (NR-56461)	EPI_ISL_7160424	Andrew S. Pekosz

Supplemental Table 2. Amino acid substitutions of Nsp12 in VOC/VOI observed at frequency ≥1%

SARS-CoV-2 Variant	SARS-CoV-2 Variant Sequences Analyzed (N, % of Total Sequences)	Nsp12 Substitution	Frequency of Substitution, % (N)
Omicron	392,056 (6.7%)	P323L	99.5 (390,020)
		F694Y	2.0 (7,822)
Delta	4,059,836 (69.5%)	P323L	99.4 (4,034,234)
		G671S	97.8 (3,968,940)
		L838I	6.8 (277,419)
		F192V	5.8 (235,631)
		F694Y	4.9 (197,489)
		R197Q	1.2 (47,122)
Alpha	1,158,351 (19.8%)	P323L	99.3 (1,149,857)
		P227L	14.2 (164,221)
Beta	35,180 (0.60%)	P323L	91.7 (37,661)
		L829F	9.0 (3,712)
		P323H	7.5 (3,075)
		Q822H	5.4 (2,198)
		A176T	2.3 (940)
Gamma	120,614 (2.1%)	P323L	99.3 (119,734)
Epsilon	44,549 (0.76%)	P323L	94.9 (42,274)
		G671V	2.1 (937)
Zeta	1,834 (0.03%)	P323L	98.2 (1,801)
		M196L	1.9 (34)
Iota	20,923 (0.36%)	P323L	99.5 (20,821)
		M601I	2.3 (485)
		V257A	2.1 (438)
		D62G	1.2 (244)
		A529V	1.2 (241)

Kappa	5,498 (0.09%)	P323L	99.5 (5,472)
		V675I	7.8 (431)
		K478N	7.1 (388)
		T26I	4.0(218)
		P809R	1.6 (88)
Lambda	6,219 (0.11%)	P323L	99.8 (6,209)
Mu	7,888 (0.14%)	P323L	98.5 (7,771)
		Y521C	21.8 (1,722)
		M629I	9.6 (756)
		T344I	2.3 (181)
		A656S	1.6 (124)
		S364F	1.4 (109)

Supplemental Table 3. Amino acid substitutions of Spike in VOC/VOI observed at frequency ≥1%

SARS-CoV-2 Variant	SARS-CoV-2 Variant Sequences Analyzed, N (% of Total Sequences)	Spike Substitutions	Frequency of Substitution, % (N)
Omicron	392,056 (6.7)	D614G	99.6 (390,638)
		H655Y	99.5 (389,923)
		N679K	99.4 (389,751)
		P681H	99.4 (389,598)
		N969K	98.3 (385,175)
		Q954H	98.0 (384,342)
		T547K	97.8 (383,385)
		D796Y	97.1 (380,607)
		A67V	96.9 (379,804)
		L981F	96.6 (378,693)
		N856K	96.5 (378,369)
		T95I	96.0 (376,297)
		HV69_70.del	95.1 (372,722)
		G142D	95 (372,462)
		YVY144_145.del	93.6 (366,879)
		G339D	92.2 (361,471)
		Q493R	88.3 (346,211)
		T478K	88.0 (345,149)
		N501Y	87.7 (343,910)
		S477N	87.7 (343,796)
		E484A	87.5 (343,201)
		Q498R	87.4 (342,647)
		Y505H	87.3 (342,159)
		G496S	86.0 (337,306)
		S375F	85.3 (334,245)
		S373P	85.2 (334,038)

		N211del	84.5 (331,246)
		L212I	84.5 (331,183)
		S371L	83.3 (326,673)
		ins214EPE	79.7 (312,289)
		N764K	73.7 (288,937)
		N440K	41.8 (163,741)
		G446S	41.5 (162,582)
		K417N	38.6 (151,376)
		R346K	23.6 (92,580)
		A701V	13.9 (54,486)
		I1081V	4.1 (15,944)
		S371F	1.6 (6,166)
		T19I	1.6 (6,106)
		D405N	1.6 (6,076)
		T376A	1.5 (6,044)
		V213G	1.5 (6,004)
		R408S	1.5 (5,968)
		A27S	1.4 (5,305)
		PPL24_26.del	1.4 (5,282)
Delta	4,059,836 (69.5)	D614G	99.7 (4,046,614)
		P681R	99.5 (4,038,886)
		T19R	98.5 (3,998,522)
		T478K	97.4 (3,955,352)
		L452R	97.2 (3,944,329)
		D950N	95.5 (3,876,829)
		FR157_158.del	91.9 (3,728,796)
		R158del	91.6 (3,718,449)
		G142D	65.5 (2,660,026)
		T95I	38.5 (1,563,622)
		A222V	10.2 (412,176)

		Y145H	3.4 (137,276)
		V1264L	2.5 (102,597)
		S112L	1.8 (71,539)
		L5F	1.4 (57,319)
		V1104L	1.4 (56,592)
		D950B	1.2 (50,256)
		Q613H	1.2 (49,816)
Alpha	1,158,351 (19.8)	D614G	99.6 (1,153,400)
		A570D	99.5 (1,152,134)
		P681H	99.3 (1,149,903)
		T716I	99.0 (1,146,565)
		S982A	98.8 (1,143,902)
		D1118H	98.7 (1,143,499)
		N501Y	97.8 (1,132,631)
		HV69_70.del	94.8 (1,098,478)
		Y144del	93.9 (1,087,059)
		K1191N	3.4 (39,741)
		L5F	3.4 (39,474)
		S98F	1.8 (20,357)
		W152R	1.5 (17,596)
		D138H	1.1 (12,137)
Beta	35,180 (0.60)	D614G	99.5 (35,003)
		A701V	99.1 (34,869)
		D80A	93.9 (33,028)
		K417N	92.6 (32,585)
		D215G	92.5 (32,557)
		N501Y	89.9 (31,628)
		E484K	89.4 (31,436)
		LAL242-244del	83.5 (29,361)
		L18F	44.1 (15,521)

		A27S	10.8 (3,786)
		T19I	2.4 (850)
		P384L	1.8 (644)
		L241del	1.7 (586)
		A899S	1.6 (575)
		L5F	1.6 (558)
		A67V	1.1 (399)
Gamma	120,614 (2.1)	D614G	99.5 (119,958)
		H655Y	98.5 (118,832)
		V1176F	97.0 (116,930)
		T1027I	96.9 (116,868)
		L18F	95.9 (115,714)
		P26S	95.7 (115,423)
		T20N	95.4 (115,087)
		K417T	95.2 (114,812)
		N501Y	95.1 (114,694)
		E484K	94.8 (114,366)
		D138Y	93.7 (112,995)
		R190S	92.9 (112,060)
		P681H	5.0 (6,063)
		A688V	2.6 (3,160)
		N679K	1.6 (1,884)
		S1252F	1.4 (1,660)
		Q675H	1.1 (1,342)
		H49Y	1.0 (1,254)
Epsilon	44,549 (0.76)	D614G	99.8 (44,472)
		L452R	97.4 (43,409)
		W152C	95.0 (42,311)
		S13I	84.0 (37,439)
		W258L	8.7 (3,871)

		P26S	5.7 (2,517)
		L5F	1.8 (819)
		S1252F	1.5 (678)
Zeta	1,834 (0.03)	D614G	100.0 (1,833)
		V1176F	97.7 (1,791)
		E484K	94.7 (1,737)
		F565L	5.8 (106)
		L5F	2.4 (43)
		T859I	1.0 (19)
		A626S	1.0 (19)
Iota	20,923 (0.36)	D614G	99.7 (20,863)
		T95I	98.4 (20,582)
		D253G	97.8 (20,471)
		L5F	95.9 (20,073)
		A701V	63.6 (13,313)
		E484K	53.3 (11,152)
		S477N	39.7 (8,314)
		Q957R	31.4 (6,570)
		L18F	3.5 (733)
		D1260N	3.1 (657)
		A845S	1.8 (375)
Kappa	5,498 (0.09)	D614G	99.8 (5,488)
		P681R	99.5 (5,470)
		E484Q	95.8 (5,266)
		L452R	95.7 (5,263)
		Q1071H	72.3 (3,973)
		E154K	67.7 (3,721)
		T95I	44.2 (2,429)
		G142D	43.4 (2,387)
		H1101D	32.9 (1,807)

		V382L	8.3 (455)
		D1153Y	8.1 (444)
		Q218H	4 (221)
		V3G	2.1 (113)
		H1101Y	1.6 (87)
		Y144del	1.5 (81)
		V1264L	1.3 (70)
		E1072K	1 (55)
		K1073R	1 (55)
Lambda	6,219 (0.11)	D614G	99.9 (6,213)
		T859N	99.1 (6,160)
		L452Q	98.2 (6,105)
		F490S	97.5 (6,063)
		G75V	94.4 (5,873)
		T76I	94.4 (5,871)
		D253N	78.4 (4,878)
		RSYLTPG.246_252.del	78.2 (4,863)
		Q675H	8.6 (534)
		I714V	4.6 (283)
		HV69_70del	3.5 (216)
		T572I	2.8 (173)
		L5F	2.2 (138)
		T63I	2.1 (130)
		G72E	1.5 (93)
		Q677H	1.5 (91)
		R21I	1.4 (88)
		A262S	1.4 (84)
		DS.253_254del	1.1 (66)
		T20I	1 (63)
Mu	7,888 (0.14)	D614G	99.8 (7,875)

		P681H	99.6 (7,855)
		R346K	99.2 (7,824)
		T95I	96.2 (7,590)
		N501Y	96.2 (7,589)
		E484K	95.8 (7,553)
		D950N	92.9 (7,324)
		Y145N	84 (6,627)
		ins143T	76.4 (6,026)
		Y144S	68.3 (5,389)
		E583D	5.3 (420)
		Y449N	5.3 (417)
		K417N	5.3 (415)
		D950B	4.8 (379)
		Y144T	4.8 (375)
		M1229I	3.4 (270)
		S939F	2.9 (230)
		ins144S	2.5 (201)
		Y144del	2.4 (193)
		Y144N	2.1 (165)
		T572I	1.9 (153)
		M1237I	1.7 (133)
		L5F	1.5 (121)

Supplemental Table 4. Amino acid substitutions of Nsp7 in VOC/VOI observed at frequency ≥1%

Variants	Sequence Count (Count/Total sequence N=5,842,948)	Nsp7 substitutions with Frequency >= 1% in the variants	Frequency of Substitution, % (N)
Omicron	392056 (6.7%)	-	
Delta	4059836 (69.5%)	-	
Alpha	1158351 (19.8%)	-	
Beta	35180 (0.60%)	L56F	2.6 (913)

Gamma	120614 (2.1%)	-	
Epsilon	44549 (0.76%)	-	
Zeta	1834 (0.03%)	L71F	99.1 (1818)
Iota	20923 (0.36%)	-	
Kappa	5498 (0.09%)	L71F	2.4 (130)
Lambda	6219 (0.11%)	-	
Mu	7888 (0.14%)	-	

Supplemental Table 5. Amino acid substitutions of Nsp8 in VOC/VOI observed at frequency ≥1%

Variants	Sequence Count (Count/Total sequence N=5,842,948)	Nsp8 substitutions with Frequency >= 1% in the variants	Frequency of Substitution, % (N)
Omicron	392056 (6.7%)	-	
Delta	4059836 (69.5%)	-	
Alpha	1158351 (19.8%)	Q24R	6.1 (70236)
		T145I	1.0 (11659)
Beta	35180 (0.60%)	-	
Gamma	120614 (2.1%)	-	
Epsilon	44549 (0.76%)	-	
Zeta	1834 (0.03%)	P133S	5.2 (95)
Iota	20923 (0.36%)	A14V	1.2 (248)
Kappa	5498 (0.09%)	-	
Lambda	6219 (0.11%)	-	
Mu	7888 (0.14%)	-	
